# Pars plana vitrectomy in progressive severe stellate non-hereditary idiopathic foveomacular retinoschisis (SNIFR): surgical outcomes and considerations for pathophysiology

**DOI:** 10.1186/s40942-025-00742-w

**Published:** 2025-10-24

**Authors:** Omar M. Moinuddin, Remo Turchetti Moraes, Beatriz Klejnberg Moraes, Eduardo Cunha de Souza, Octaviano Magalhaes Junior, Nadyr Damasceno, Hélio P. Primiano Júnior, Antonio Capone Jr., Antonio Marcelo B. Casella, Tamer H. Mahmoud

**Affiliations:** 1 Associated Retinal Consultants, P.C., Royal Oak, MI USA; 2 Oakland University William Beaumont School of Medicine, Rochester, MI USA; 3 Brazilian Institute of Ophthalmology, Rio de Janeiro, Brazil; 4 Ophthalmology Division, São José do Rio Preto Medical School, São José do Rio Preto, Brazil; 5 Department of Ophthalmology, Universidade Federal de São Paulo, São Paulo, Brazil; 6 Department of Ophthalmology, Hospital Naval Marcilio Dias, Rio de Janeiro, Brazil; 7 Department of Ophthalmology, Universidade Estadual de São Paulo, São Paulo, Brazil; 8 Department of Surgery, Universidade Estadual de Londrina, Londrina, Brazil

**Keywords:** Foveomacular schisis, Retinoschisis, Idiopathic, Pars plana vitrectomy, Hyaloid, Internal limiting membrane, Membrane peel, Macular thickness

## Abstract

**Background:**

To report the clinical course and outcomes of a surgical approach for progressive severe stellate non-hereditary idiopathic foveomacular retinoschisis (SNIFR) using pars plana vitrectomy (PPV).

**Methods:**

Multi-center, consecutive, interventional case series. Patients with a diagnosis of SNIFR presenting with progressive loss of vision between January 1, 2017 and January 1, 2023. Evaluation of ophthalmologic findings and multimodal ocular imaging at the time of diagnosis, surgical procedure, and of visual and anatomic outcomes postoperatively. The main outcome measures evaluated include best corrected visual acuity (BCVA), central macular thickness (CMT), and findings on optical coherence tomography (OCT).

**Results:**

Seven patients diagnosed with SNIFR were included. Median age in years at the time of diagnosis was 64 (range, 46–77). Four patients were female, and three were male. Genetic testing for mutations in retinoschisin 1 (RS1) and for other inherited conditions associated with foveomacular retinoschisis was negative. All patients demonstrated progressive and severe retinoschisis, as well as worsening vision loss and metamorphopsia when managed conservatively. PPV was performed and revealed anomalously broad and dense adherence of the posterior hyaloid in all eyes. The internal limiting membrane (ILM) was peeled in all but one case. Median BCVA at baseline measured 20/50, and declined to 20/70 at the time of surgery. Median preoperative CMT measured 561 μm, with OCT demonstrating prominent retinoschisis of the outer plexiform and outer nuclear layers. All eyes demonstrated postoperative resolution of retinoschisis and subretinal fluid, with corresponding improvements in both BCVA and subjective central visual distortion up to six months after surgery. BCVA for the entire cohort improved to a median of 20/30, and with a corresponding decrease in CMT to a median of 240 μm.

**Conclusion:**

PPV is an effective surgical intervention resulting in anatomic resolution of retinoschisis and improved functional vision in eyes with progressive and severe SNIFR.

## Background

Initially described in 2014, stellate nonhereditary idiopathic foveomacular retinoschisis (SNIFR) is a distinct clinical entity characterized by a stellate pattern of foveomacular schisis in the absence of identifiable inherited or acquired causes [1]. As a diagnosis of exclusion, SNIFR is established only after thorough investigation to rule out other known etiologies of foveomacular retinoschisis. These include congenital X-linked retinoschisis (CXLRS), enhanced S-cone syndrome (ESCS), Goldmann-Favre syndrome (GFS), myotonic dystrophy, structural anomalies of the optic disc, glaucoma, myopic traction maculopathy, and exposure to niacin and taxane derived medications [2, 3].

The disease is classically indolent, with a vast majority of patients preserving a best-corrected visual acuity (BCVA) of 20/40 or better. However, a subset of eyes develop significant vision loss due to progressive schisis of the outer plexiform (OPL), Henle fiber layer (HFL), outer nuclear layer (ONL), as well as the accumulation of subfoveal fluid [4–7]. Given its generally good visual prognosis, SNIFR is often observed. However, some have performed surgery with variable results. As with its pathophysiology, the management of SNIFR remains poorly understood and current treatment options are not well defined in the literature. Moreover, consensus on which eyes to treat and when to intervene is lacking.

Herein, we present a multi-center series evaluating the clinical course, surgical management, and outcomes of patients with progressive and severe SNIFR managed using pars plana vitrectomy (PPV). To the best of our knowledge, this study represents the largest case series and longest documented follow-up of eyes with SNIFR undergoing surgical treatment reported to date. We further present a comprehensive characterization of the disease and surgical technique, as well as an updated review of the literature on the pathophysiology and modern management.

## Methods

Research conducted was in compliance with the Health Insurance Portability and Accountability ACT (HIPAA) and the Declaration of Helsinki, while abiding to all regional, national, and international laws of the institutions involved in this study. Informed patient consent was obtained for genetic testing, and every effort was made by the investigators to protect the rights of patients and their respective families during the course of this investigation. This study is in concordance with the tenets of the ethics committee of each contributing center, and was approved by the Western Institutional Review Board.

In each case, a diagnosis of SNIFR was made based on a combination of clinical history, ophthalmoscopy, review of multimodal ocular imaging, axial length measurement, and genetic testing to rule out inherited conditions associated with foveomacular retinoschisis. Patients presenting between January 1, 2017 and January 1, 2023 were selectively included in this investigation. Collected data included age, anatomic sex, past medical and family history, results of genetic testing and pedigree analysis, findings on ophthalmologic examination and operative reports, treatment outcomes, and analyses of ocular imaging.

## Results

A total of seven patients (seven eyes) were included in this study, of which three (42.9%) were males and four (57.1%) were females. All cases were unilateral. The median length of follow-up was 24 months, (range 18–68 months) for the cohort (Table 1).

**Table 1:** Patient demographics

**Sex**	
Female, no (%)	4 (57.1%)
Male, no (%)	3 (42.9%)
**Age**,** years**	
Median, (range)	64, (46–77)
**Length of follow-up**,** months**	
Median, (range)	24, (18–68)

Patient sex, age, and median length of follow-up in months

**Fig. 1:**
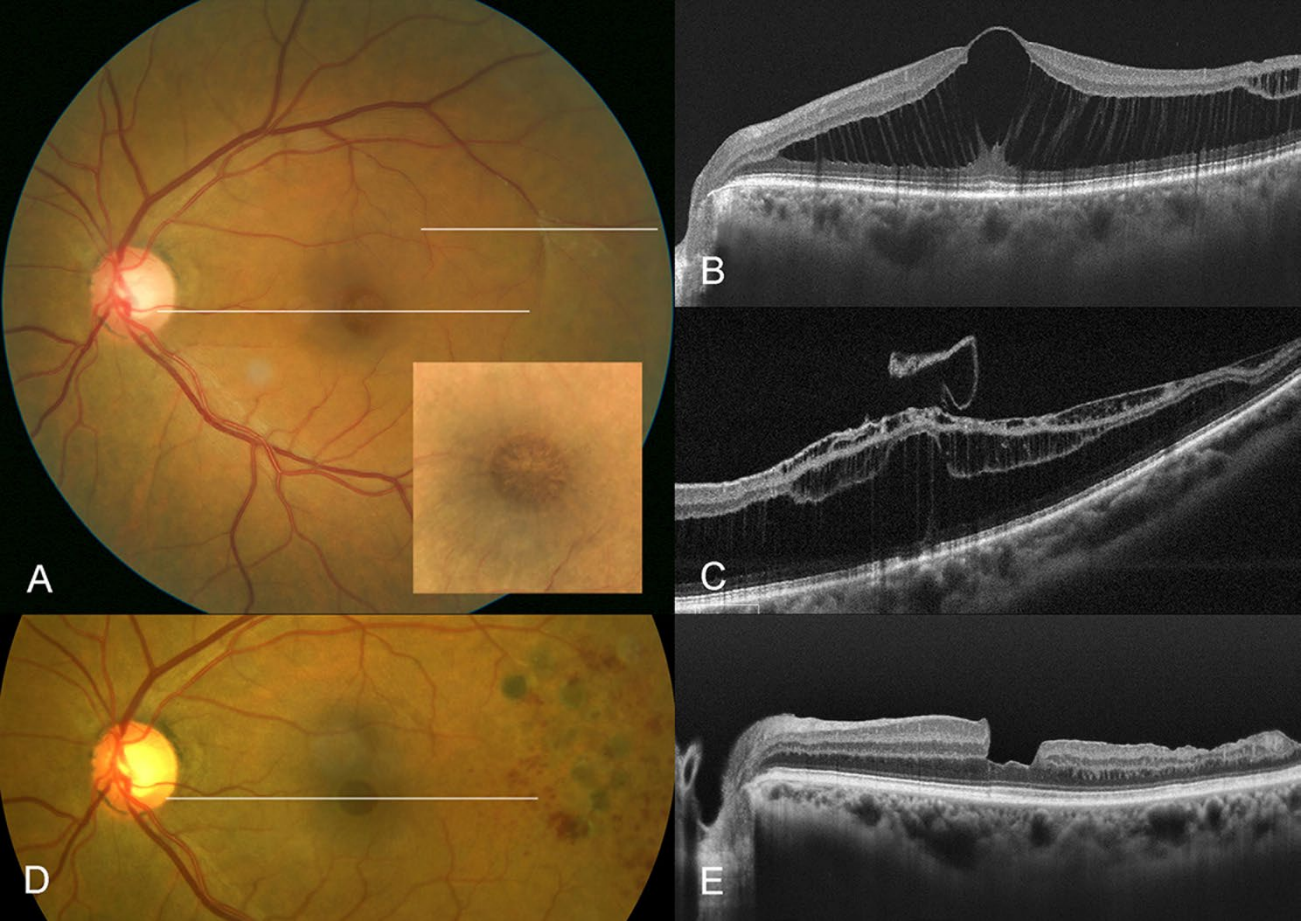
Multimodal imaging of patient 4. **A.** Color fundus photography at presentation. Spoke-like radial cysts with a stellate appearance of the macula are noted (inset). An anomalous temporal vitreoretinal adhesion can be noted temporal to the macula. **B**,** C.** OCT at presentation. The outer plexiform layer schisis with cystoid spaces in the foveal area, and a temporal inner nuclear layer splitting with an anomalous vitreous adhesion are observed. **D.** Six-month postoperative color fundus photography. **E.** Six-month postoperative OCT. Foveomacular retinoschisis is resolved

All eyes exhibited a blunted foveal light reflex with a subtle starburst pattern radiating from the fovea. Careful examination of the retinal periphery with indirect binocular ophthalmoscopy with scleral depression and ocular ultrasound revealed a notable absence of peripheral retinoschisis. Intraocular pressure was within normal limits, and Ishihara color plate testing was unremarkable. Ocular imaging demonstrated alternating areas of spoke-like hyporeflectivity and hyperreflectivity on IR, retinoschisis of the OPL, HFL, and to a comparatively lesser degree of the ONL on OCT (Fig. 1), and no defined hyperfluorescence consistent with staining or leakage on FA. There was no evidence of myopic degeneration, lamellar macular or full thickness macular hole, epiretinal membrane, vitreomacular traction, or optic pit or other structural anomaly of the optic nerve on ophthalmoscopy or imaging.

All patients denied experiencing nyctalopia, hemeralopia, constriction of visual field, systemic symptoms of myotonia or muscle weakness, and reported no known family history of CXLRS, ESCS, GFS, myotonic dystrophy, optic neuropathy, or visual impairment from an early age. Genetic testing including for retinoschisin 1 (RS1) was negative. Medical history was unrevealing for malignancy, autoimmune disease, and for current or past use of taxane or niacin based medications. Past ophthalmic history was also unremarkable, with the exception of uncomplicated cataract surgery performed in the affected eye in four patients (57.1%).

The median age in years at the time of SNIFR diagnosis was 64 (range, 46–77). Median BCVA at presentation measured 20/50 (range, 20/25 − 20/100). All eyes exhibited significant retinoschisis of the OPL and HFL, milder retinoschisis of the ONL, and subfoveal fluid was present in three eyes (42.9%) at initial presentation. Two patients (28.6%) remained asymptomatic and were managed conservatively. Five patients (71.4%) reported symptomatically decreased vision and progressive metamorphopsia, and were treated with topical 2% dorzolamide hydrochloride ophthalmic solution three times daily with no resulting improvement in functional vision. All eyes exhibited progression of retinoschisis over a subsequent period of twelve months. At the time of surgery the median BCVA for the entire cohort declined to 20/70 (range, 20/30 − 20/250), and all patients reported progressively worsening central metamorphopsia. Due to the continued progression of severe retinoschisis, subretinal fluid, and worsening vision, we opted to perform pars plana vitrectomy (PPV) with elevation and removal of the hyaloid membrane. Written informed consent for surgery was obtained from all patients, and the operations were performed without complication by experienced surgeons at their respective institutions.

All surgeons performed 25-gauge PPV, with intravitreal triamcinolone acetonide used to enhance visualization and facilitate mechanical elevation of the posterior hyaloid membrane. Notably, all surgeons observed the posterior hyaloid to demonstrate broad adherence to the retinal surface. Intraoperative findings were consistent across all seven eyes, with anomalous vitreoschisis warranting meticulous dissection of distinct layers of hyaloid and rendering surgical induction of PVD particularly challenging. Following successful lifting of the hyaloid membrane, the residual cortical vitreous was noted to be densely adherent and tightly integrated to the inner retina. Accordingly, careful peeling of the internal limiting membrane (ILM) was performed to further relieve this residual traction exerted by the cortical vitreous in all but one case (Case 2).

Brilliant Blue G (Dutch Ophthalmic Research Center, Zuidland, Netherlands) or Indocyanine Green (Akon Pharmaceuticals, Lake Forest, USA) was used in three eyes each to stain and enhance visualization of the ILM, based on individual surgeon’s preference. Similar to the posterior hyaloid, the ILM demonstrated anomalous coherence and posed significant challenges to peeling requiring careful and meticulous dissection. The ILM was ultimately successfully peeled up to near the arcades, corresponding to the broad-based attachment of the posterior hyaloid. A complete fluid-air exchange was performed in all eyes. A dilute gas tamponade was instilled in the six eyes that underwent PPV with subsequent ILM peeling, with sulfur hexafluoride (SF6) used in two eyes and perfluoropropane (C3F8) used in four eyes.

**Fig. 2:**
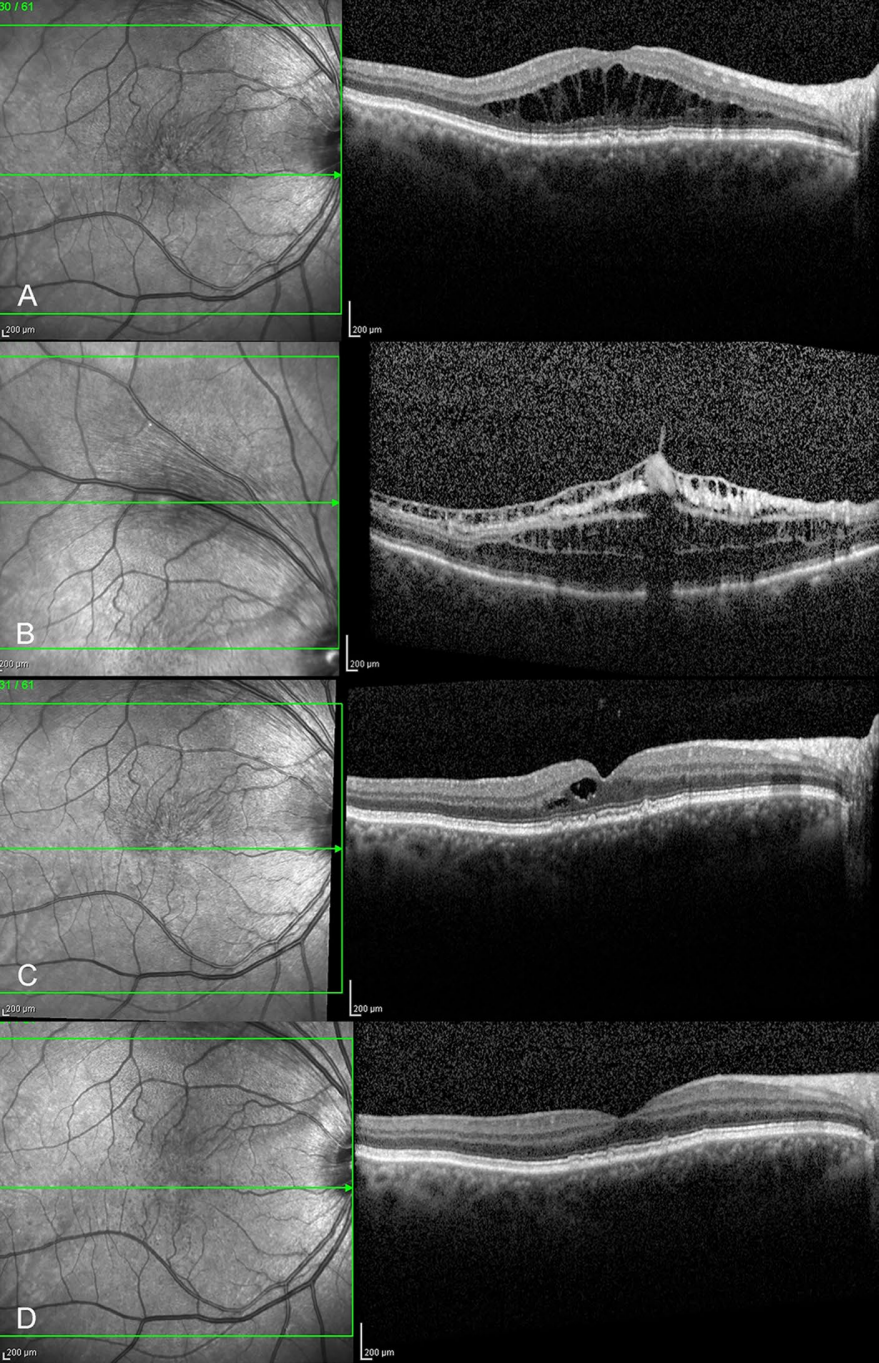
OCT imaging of patient 3. **A**,** B.** Preoperative. The outer plexiform layer schisis with cystoid spaces in the foveal area, and a temporal inner nuclear layer splitting with an anomalous vitreous adherence near the superior temporal arcade are observed. **C.** One month surgery, improving intraretinal schisis. **D.** Six months after surgery, foveomacular retinoschisis is resolved

Postoperatively all seven eyes demonstrated gradual improvement, and ultimately resolution of retinoschisis over a period of four to six months (Fig. 2). Subfoveal fluid resolved in all eyes. OCT demonstrated a significant reduction in central macular thickness (CMT) from a preoperative median of 561 μm (range, 493–795 μm) to a postoperative median CMT of 240 μm (range, 192–307 μm). All eyes demonstrated a gradual improvement in vision up to a period of six months following surgery (Table 2). At most recent follow-up postoperative vision improved to a median BCVA 20/30 (range, 20/20–20/50), with all patients reporting a marked improvement in the degree of metamorphopsia. All eyes were pseudophakic.

**Table 2:** Clinical characteristics and surgical outcomes

Case	Age, years	Sex	Laterality	Presenting BCVA	Preoperative BCVA	Preoperative CMT	BCVA Postoperative Month 1	BCVA Postoperative Month 3	BCVA Postoperative Month 6	CMT Postoperative Month 6	Treatment Response
1	74	Male	OS	20/50	20/250	602 μm	20/125	20/50	20/50	307 μm	Improved foveomacular schisis, reduced CMT, resolved SRF
2	77	Male	OD	20/30	20/60	517 μm	20/50	20/30	20/30	240 μm	Improved foveomacular schisis, reduced CMT
3	62	Female	OD	20/70	20/70	557 μm	20/80	20/30	20/30	250 μm	Improved foveomacular schisis, reduced CMT
4	64	Female	OS	20/40	20/80	795 μm	20/40	20/40	20/40	282 μm	Improved foveomacular schisis, reduced CMT
5	61	Female	OD	20/25	20/30	581 μm	20/20	20/20	20/20	239 μm	Improved foveomacular schisis, reduced CMT
6	66	Female	OS	20/50	20/50	493 μm	20/20	20/20	20/20	202 μm	Improved foveomacular schisis, reduced CMT, resolved SRF
7	46	Male	OD	20/100	20/100	561 μm	20/80	20/60	20/30	192 μm	Improved foveomacular schisis, reduced CMT, resolved SRF

Anatomic and visual outcomes following operation

Abbreviations: oculus dexter (OD); oculus sinister (OS); best-corrected visual acuity (BCVA); central macular thickness (CMT); subretinal fluid (SRF).

## Discussion

First described by Ober et al. in 2014, SNIFR is a rare idiopathic condition characterized by radial spoke-like schisis of the outer retina within the parafovea [1]. SNIFR is a diagnosis of exclusion that occurs in the absence of other acquired or inherited forms of foveomacular schisis, and comprises up to 2% of such cases [8]. The condition is broadly defined as preferential splitting of the HFL along the posterior margin of the OPL, often with comparatively lesser schisis of the ONL. In contrast to CXLRS that is congenital, demonstrates bi-macular involvement, frequently involves the retinal periphery, and assumes an x-linked pattern of inheritance, SNIFR is classically diagnosed in middle aged adults, often unilateral, and typically presents without peripheral retinoschisis [1, 2, 9]. Moreover, similar to our findings, the literature demonstrates SNIFR occurs in women more often than in men [1, 6]. 

SNIFR is a generally indolent disease process with the majority of patients maintaining vision better than or equal to 20/40 [3, 10]. Yet, there is a notable number of eyes with significant vision loss and symptomatic metamorphopsia. These eyes tend to demonstrate more extensive stellate retinoschisis of the HFL, often also with involvement of the ONL, and the presence of subretinal fluid. While treatment in such patients is imperative, our current understanding of both the pathophysiology and management of this condition remains obscure, and there exists limited data on the outcomes of eyes with progressive severe SNIFR.

A review of the current literature suggests the development of SNIFR is attributed to continued tractional forces exerted by the posterior hyaloid, [4, 8, 11, 12] particularly on the HFL which lacks the structural support provided by local vessels [3, 6, 13]. Indeed, prior studies of retinal ultrastructure have demonstrated the preferential location of retinoschisis in SNIFR aligns with pillars of avascular tissue made up of muller cells and photoreceptor axons within the HFL [3, 14, 15]. This pattern of stellate retinoschisis localized to the avascular HFL is reaffirmed by studies of OCT and OCT-angiography in eyes with SNIFR demonstrating overlap of schisis cavities with areas of attenuated vascular signal [6, 13]. More recent research further demonstrates the role of posterior hyaloidal traction in this condition. In the largest reported cohort of SNIFR to date, Bloch et al. documented incomplete separation of the posterior hyaloid in 86% of affected eyes compared to 42% in unaffected fellow eyes. Hyaloidal attachment in these eyes was observed to be anomalously broad, with several eyes exhibiting retinoschisis spanning contiguously from the fovea to the limits of the macular cube scan on OCT [8]. 

Coinciding with our described intraoperative observations, Feo et al. more recently described the use of multimodal ocular imaging to demonstrate the anomalously broad posterior vitreoretinal adhesion and/or traction especially in the mid-periphery in eyes with SNIFR [16]. The investigators describe mid-peripheral retinoschisis (MPRS) can progress to SNIFR over multiple years, and that SNIFR with MPRS may remain stable, spontaneously resolve over time, or become further complicated by a mid-peripheral inner retinal microvasculopathy. Feo et al. coined the term “Central Anomalous Retinoschisis with Mid-Peripheral Traction (CARPET)” to describe a severe variant of this disease driven by prominent vitreoretinal adhesion, and characterized by a triad of (1) SNIFR with significant mid-peripheral traction, (2) central neurosensory detachment, and (3) outer lamellar macular hole. While prominent mid-peripheral retinoschisis and associated microangiopathy were not observed in the cases of progressive SNIFR described herein, future studies incorporating preoperative screening with ultra-widefield OCT and intraoperative OCT may reveal subtle manifestations of these features and further elucidate their clinical and prognostic relevance.

The role of anomalous traction from the posterior hyaloid in the pathophysiology of this condition is further substantiated by reports of spontaneously resolving foveomacular schisis and improved vision following release of vitreomacular adhesion in SNIFR eyes. Nogueira et al. initially reported this observation in a 67-year-old woman with SNIFR exhibiting complete resorption of fluid and resolution of foveomacular schisis six months following release of VMA [11]. Liu et al. reported a similar case of asymmetric SNIFR in which one eye exhibited resolution of central macular schisis and improved vision six months after spontaneous resolution of VMA, while the fellow eye with persistently worsening disease exhibited resolution of schisis and improved vision only after vitrectomy surgery to elevate the posterior hyaloid [12]. Moraes et al. first described a surgical approach to SNIFR using PPV with ILM peeling and gas tamponade, and reported gradual regression of schisis cavities and macular thickness, resorption of subretinal fluid, and reapproximation of the outer retinal layers on OCT occurring up to twelve months after surgery [4]. Feo et al. similarly performed PPV and mechanical lifting of the posterior hyaloid in a patient with CARPET and observed the progressive resolution of SNIFR and resorption of subfoveal fluid, along with corresponding improvement in BCVA from 20/200 to 20/25 over a period of twelve months. In contrast, two other eyes in this series did not undergo surgery and experienced worsening central inner and middle retinoschisis, subfoveal detachment, and ultimately worsening of functional vision [16].

Non-surgical treatments for eyes with vision loss due to SNIFR have been shown to be comparatively less effective. Ajlan et al. initially noted temporary improvement in macular thickness with the use of topical carbonic anhydrase inhibitor (CAI) therapy, but reported recalcitrant schisis and vision loss when the medication was discontinued [7]. Schildroth et al. subsequently reported using topical CAI in three eyes with SNIFR of varying severities of retinoschisis, and noted no improvement in vision or anatomy despite up to 19 months of treatment [6]. Treatment with intravitreal anti-vascular endothelial growth factor has also been proposed, particularly in eyes with co-morbid neovascular age-related macular degeneration. However, these studies predictably demonstrated regression of choroidal neovascularization and exudation, but with otherwise unaltered and persistent retinoschisis [5, 6]. In our cohort, all eyes were initially managed with observation alone. Topical CAI therapy was trialed in four patients experiencing progressive vision loss and metamorphopsia, but no improvement was observed and all eyes experienced worsening foveomacular schisis.

**Fig. 3:**
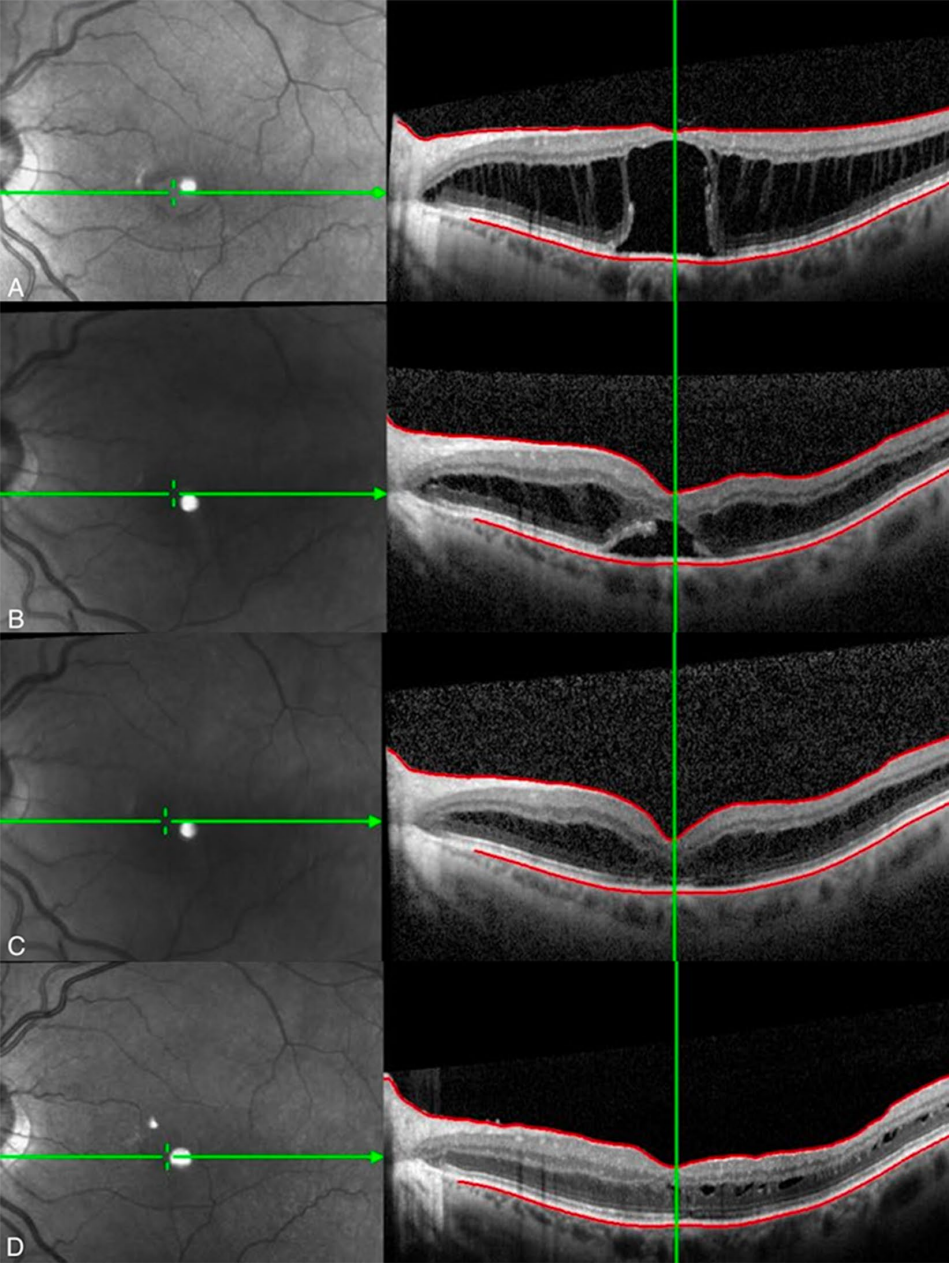
OCT imaging of patient 1. **A.** Preoperative. Extensive retinoschisis and central neurosensory detachment. **B.** One month after surgery, significant improvement in retinoschisis and subretinal fluid. **C.** Three months after surgery, resolution of subretinal fluid and continued improvement in retinoschisis. **D.** Five months after surgery, subretinal fluid remains resolved and with near complete resolution of retinoschisis

Given the worsening retinoschisis and continued decline in vision with conservative medical management, we imperatively escalated treatment to a surgical approach with PPV. The use of IVTA enhanced visibility of the vitreous to facilitate elevation of the posterior hyaloid and revealed a significant presence of vitreoschisis. Remnant cortical vitreous densely adherent to the ILM was observed. Moreover, this observation is supported by ultrastructural analysis of dissected retina that has shown cortical gel frequently remains attached to the ILM after both surgically induced and spontaneous posterior vitreous detachment, and that eyes with vitreomacular pathology exhibit comparatively more severe fibrocellular proliferation at the vitreoretinal interface [17–19]. Accordingly, the ILM was subsequently peeled in six of the seven eyes (85.7%) to ensure complete removal of the posterior vitreous. We further theorize membrane peeling eliminates potential contributive tangential traction from stiffening of the ILM in cases of more advanced SNIFR, as is well established in other progressive disorders of the vitreomacular interface [20, 21]. All eyes demonstrated progressive improvement in retinoschisis and resolution of subretinal fluid (Fig. 3), with a median improvement in CMT of 307 μm (range, 277–513 μm). All patients demonstrated significant improvement in BCVA and reported a reduction in central metamorphopsia up to six months following the operation. In eyes with progressive and severe SNIFR warranting surgical intervention, we observe recovery of functional vision following resolution of retinoschisis and subretinal fluid. Both Moraes et al. and Feo et al. similarly observed this temporal relationship in which anatomic reconstitution precedes visual improvement postoperatively [4, 16]. Furthermore, this observed delay between anatomic and visual improvement remains consistent with our surgical experience managing other more conventional macular diseases with well-established pathophysiology attributed to an anomalous hyaloidal-macular interface such as full-thickness macular hole.

The slow but complete resolution of retinoschisis after the release of the traction through pars plana vitrectomy seems interesting and puzzling. Similar outcome cannot be achieved in XL-retinoschisis. One would wonder about the role of Retinoschisin protein and its ability to reconstruct the retina. One would expect the release of traction to stop the progression of retinoschisis and recovery of foveal anatomy to baseline SNIFR, prior to the progression with vision loss and metamorphopsia. The lack of absence of the protein may explain the complete resolution of retinoschisis, as the retina is able to reconstruct internally.

Limitations to this study include the rarity of this condition, the retrospective nature of the investigation, small sample size, and the non-randomized selection of patients. Genetic testing was limited only to patients, and did not include family members. Furthermore, operative technique was not standardized among the various surgeons.

## Conclusions

In summary, we report a retrospective interventional case series of seven patients with progressive severe SNIFR who were managed with surgical intervention using PPV. We report consistent intraoperative findings of anomalously broad and dense adherence of the posterior hyaloid, as well as significant vitreoschisis across all eyes. We further describe a surgical approach using PPV to mechanically elevate the posterior hyaloid, and with careful consideration given to peeling the ILM to further relieve traction exerted by remnant cortical vitreous and stiffened ILM that may exist in eyes with more advanced progressive SNIFR. We demonstrate successful resolution of retinoschisis and significant improvement in functional vision occurring up to six months following surgery in all cases.

We further provide an updated review of the current literature to describe the pathophysiology of this rare condition, which is based on anomalous hyaloidal traction resulting in retinoschisis of the fragile HFL/OPL that is inherently devoid of structural support from local retinal vasculature. The consistent intraoperative findings and favorable outcomes reported by multiple surgeons lend further evidence to support this pathophysiology, and to establish an evidence-based management approach for eyes with vision loss due to progressive and severe SNIFR. The experience in this multi-center series confirms that small gauge vitrectomy is a safe, targeted, and effective treatment option for this rare yet potentially progressive and sight-threatening condition.

## Data Availability

All data generated and analyzed during this investigation are included in this published article.

## Abbreviations

SNIFR: Stellate Nonhereditary Idiopathic Foveomacular Retinoschisis
BCVA: Best–Corrected Visual Acuity
OPL: Outer Plexiform Layer
ONL: Outer Nuclear Layer
PPV: Pars Plana Vitrectomy
HIPAA: Health Insurance Portability and Accountability Act
CXLRS: Congenital X–linked Retinoschisis
ESCS: Enhanced S–Cone Syndrome
GFS: Goldmann–Favre Syndrome
IR: Infrared Imaging
OCT: Optical Coherence Tomography
FA: Fluorescein Angiography
ILM: Internal Limiting Membrane
SF_6_: Sulfur Hexafluoride
C_3_F_8_: Perfluoropropane
CMT: Central Macular Thickness
HFL: Henle Fiber Layer
CARPET: Central Anomalous Retinoschisis with Mid–Peripheral Traction
VMA: Vitreomacular Adhesion
IVTA: Intravitreal Triamcinolone Acetonide
CAI: Carbonic Anhydrase Inhibitor
